# Measuring psychological trauma after spinal cord injury: Development and psychometric characteristics of the SCI-QOL Psychological Trauma item bank and short form

**DOI:** 10.1179/2045772315Y.0000000022

**Published:** 2015-05

**Authors:** Pamela A. Kisala, David Victorson, Natalie Pace, Allen W. Heinemann, Seung W. Choi, David S. Tulsky

**Affiliations:** 1 Department of Physical Therapy, College of Health Sciences, University of Delaware, Newark, DE, USA; 2 Department of Medical Social Sciences, Northwestern University Feinberg School of Medicine, Chicago, IL, USA; 3 Rehabilitation Institute of Chicago, Chicago, IL, USA; 4 McGraw-Hill Education CTB, Monterey, CA, USA; 5 Kessler Foundation Research Center, West Orange, NJ, USA

**Keywords:** Psychological Trauma, Spinal Cord Injuries, Patient Outcomes Assessment, Quality of Life, Psychometrics

## Abstract

**Objective:**

To describe the development and psychometric properties of the SCI-QOL Psychological Trauma item bank and short form.

**Design:**

Using a mixed-methods design, we developed and tested a Psychological Trauma item bank with patient and provider focus groups, cognitive interviews, and item response theory based analytic approaches, including tests of model fit, differential item functioning (DIF) and precision.

**Setting:**

We tested a 31-item pool at several medical institutions across the United States, including the University of Michigan, Kessler Foundation, Rehabilitation Institute of Chicago, the University of Washington, Craig Hospital and the James J. Peters/Bronx Veterans Administration hospital.

**Participants:**

A total of 716 individuals with SCI completed the trauma items

**Results:**

The 31 items fit a unidimensional model (CFI=0.952; RMSEA=0.061) and demonstrated good precision (theta range between 0.6 and 2.5). Nine items demonstrated negligible DIF with little impact on score estimates. The final calibrated item bank contains 19 items

**Conclusion:**

The SCI-QOL Psychological Trauma item bank is a psychometrically robust measurement tool from which a short form and a computer adaptive test (CAT) version are available.

## Introduction

Most traumatic spinal cord injuries (SCIs) result from potentially life-threatening events, including motor vehicle crashes, followed by acts of violence (e.g. gunshot wound), falls, and diving.^1,2^ Given the traumatic nature of SCI, including the event causing the injury, paralysis and loss of sensation, and sometimes witnessing another person's death or injury, increased symptoms of acute and post-traumatic stress are a not unusual consequence. The *Diagnostic and Statistical Manual of Mental Disorders*, Fifth Ed. (DSM-5) identifies individuals with SCI as being at high risk for post-traumatic stress disorder (PTSD).^3^

The DSM-5 criteria for PTSD require a history of exposure to a traumatic event that involves the perceived or actual threat of death or serious bodily injury to oneself or others (Criterion A). PTSD is characterized by intrusive memories of the event through flashbacks, nightmares, or frightening thoughts (Criterion B); persistent affective and behavioral avoidant and numbing responses (e.g. avoiding thoughts, feelings, activities, locations that are associated with the event) (Criterion C); negative alterations in cognition or mood not previously experienced (Criterion D); and manifestations of hyper-arousal (e.g. difficulty falling asleep, irritability, reckless behavior) (Criterion E). A PTSD diagnosis requires this constellation of symptoms to develop within one week of the event, persist over one month (Criterion F), and cause significant emotional distress and functional impairment (Criterion G).^3^

Prevalence estimates of PTSD following SCI vary widely. Kennedy and Duff's review found that posttraumatic stress is common, ranging from 10–40%.^4^ Two Danish studies reported PTSD prevalence of 7–20% in a sample of 69 adults with paraplegia and tetraplegia averaging 19 years since injury.^5,6^ In a study of adults (*n* = 85) in the United Kingdom whose injuries were more recent (≤6 months), 14% demonstrated high levels of posttraumatic stress.^7^ Among 125 American veterans with SCI, Radnitz *et al*. reported that 12% met the diagnosis criteria for PTSD at the time of assessment and 29% met the criteria at some point post-injury.^8^ Within pediatric-onset SCI samples, studies report PTSD rates ranging from 25%^9,10^ to 33%.^11^ Conversely, other research suggests a SCI-PTSD prevalence that is comparable to the general population (6% to 8%).^12–14^ Variability in samples, methods, and measures likely accounts for this variation.

Pre-injury disposition, trauma-related factors, and emotional processing of the traumatic event may contribute to PTSD symptoms.^15–17^ These factors can be grouped into pre-injury, peri-injury and post-injury factors.^18^ Pre-injury factors, such as sociodemographic, physiological, and psychological characteristics may contribute to how individuals react to SCI. Younger age,^6,7^ female sex,^7^ single marital status,^5,7^ more education,^19^ and greater trauma exposure^17,20^ before SCI may predispose and influence responses to trauma. Individuals whose trauma is characterized by greater violence (e.g. gunshot, interpersonal violence) exhibit higher PTSD prevalence than those who were injured in motor vehicle crashes.^11^ Post-traumatic factors that contribute to PTSD symptoms include negative cognitions and dispositional factors, time since injury,^16,21^ and self-attributions of responsibility for injury.^22^ There may be a paradoxical association between injury level and PTSD vulnerability, with higher PTSD incidence in individuals with paraplegia rather than tetraplegia.^8,16^ There may be a curvilinear relationship between SCI disability and stress level, such that those with the most and least disability report the highest levels of stress.^17^ PTSD symptom severity typically attenuates with time.^2,16^

The psychological consequences of trauma have important clinical implications. PTSD symptoms are often persistent if left untreated and are associated with an elevated risk of morbidity. PTSD symptoms are correlated with somatic problems, anxiety, depression,^7,12,16,23^ social isolation,^24^ substance abuse, and suicide.^25^ Individuals with SCI may be at high risk for mental illness because of the negative consequences of SCI on physical and social functioning;^26–28^ in fact, elevated rates of mental illness have been observed in individuals with SCI.^29^ Identifying risk factors for PTSD or subclinical manifestations of pervasive psychological consequences of trauma is important to prevent, monitor, and treat PTSD.

A variety of assessment tools have been developed to screen for PTSD, to assess symptom severity, and diagnose the disorder. The Clinician-Administered PTSD Scale^30^ and the Structured Clinical Interview for DSM-IV PTSD module^31^ are regarded as the gold standards for diagnosis.^32,33^ Use of these measures is limited in clinical and research settings due to their length and training requirements. Del Vecchio *et al*. published a comprehensive review of peer-reviewed, self-report PTSD assessment tools.^32^ The research team identified 41 English-language instruments, including the Harvard Trauma Questionnaire (40 items),^34^ Impact of Event Scale-Revised (22 items),^35^ PTSD checklist (17 items),^36^ and the Trauma Screening Questionnaire (10 items).^37^ The lack of a common measurement metric makes it difficult to compare prevalence data and treatment outcomes across studies. The complex nature of psychological trauma experienced by persons with SCI creates a need for a self-report instrument with content specificity and sensitivity to change.

Computer adaptive testing (CAT) allows administration of brief instruments that are reliable and sensitive. CAT relies on item response theory (IRT) methods in which a computer algorithm sequentially selects a small number of items that maximizes test information and can discriminate symptom severity over time and between individuals.^38^ As part of a larger project that developed IRT-based quality of life instruments for persons with SCI, we developed an item bank to detect and track psychological trauma in patients with SCI.

As described by Tulsky *et al*. in the introductory paper of this special issue,^39^ our group has worked to unify diverse aspects of quality of life following traumatic SCI using the Patient-Reported Outcomes Measurement Information System (PROMIS)^40^ and Neuro-QOL^41^ approach. The purpose of this paper is to describe the development, psychometric analysis, and validation of the SCI-QOL Psychological Trauma item banks and short form.

## Methods

This study was approved by all participating sites' Institutional Review Boards. In brief, we developed and refined a psychological trauma item pool then administered the items to a large sample of people with SCI by in-person or telephone interviews. We describe the steps below; Tulsky *et al*. provides a detailed description of the methods.^42^

### Development of a Psychological Trauma item pool

We began by locating and classifying items from focus groups conducted with SCI patients and providers.^43^ Using the transcripts, we wrote 9 items, which we reviewed and modified as a group (authors DV, DT, PK). For example, for the patient statement, ‘*The other day we saw a huge accident right in front of me, and my own accident flashed before my eyes…it really freaked me out and I couldn't get in a car for a while after that,*’ we wrote items such as ‘I avoided reminders of how my injury occurred’ and ‘I was nervous when something reminded me of the accident.’ We used another patient's statement, ‘*It*’*s a feeling like being trapped…a prisoner in your own body*’ to write the item, ‘I felt trapped.’

One of the project investigators (author DV) had recently developed a measure of injury distress, which included a relevant post-traumatic stress scale. With minor modifications (e.g. changing ‘accident’ to ‘injury,’ and tense, i.e. present to past tense), we were able to include 16 Injury Distress Index^44^ post-traumatic stress items.

From this initial set of 25 Psychological Trauma items, we removed 6 redundant items and wrote an additional 12 items to fill gaps in what we anticipated would be the item hierarchy. Next, we completed cognitive interviews with 5 individuals with SCI.^45^ They read and answered each item, then described the thought process they used to answer the statement. We asked them to report any problems understanding the items. No items needed to be removed or modified based on cognitive interviews. Then, we reviewed items for translatability^46^ and reading level.^47^ We modified 4 items following the translatability review. For example, we modified ‘upsetting thoughts about my injury popped into my mind’ to ‘I had upsetting thoughts about the event of my injury.’ All items were written at or below a 5^th^ grade reading level.

### Calibration study participants and data collection procedures

We administered the 31-item item pool as part of a multisite calibration study (sites included the University of Michigan, Kessler Foundation, Rehabilitation Institute of Chicago, the University of Washington, Craig Hospital and the James J. Peters/Bronx Veterans Administration hospital), in which other item pools of health-related quality of life (HRQOL) items were also administered. The calibration sample was comprised of 716 individuals with traumatic SCI, all of whom were older than 18 years of age and could read and understand English. Injury level and severity (i.e. most recent American Spinal Injury Association Impairment Scale rating) were documented through medical record review. To ensure heterogeneity, the sample was stratified by diagnosis (paraplegia versus tetraplegia), severity (complete injury vs. incomplete injury), and time since injury (<1 year post injury, 1–3 years post injury, and >3 years post injury). We did not specifically exclude individuals on the basis of any concomitant injuries or conditions.

#### Data analyses

The psychometric analysis included confirmation of unidimensionality, estimation of IRT parameters using a graded response model, and examination of differential item functioning (DIF). Confirmatory factor analysis (CFA) was used to test model fit using the following standards: CFI >0.90; RMSEA < 0.08, good: CFI > 0.95, RMSEA < 0.06, excellent. Significant loadings on the single factor (values >0.30) and local item dependence (residual correlations >|0.20|) were also examined. DIF was examined using *Lordif* for six categories: age (≤49 vs. ≥50), sex (male vs. female), education (some college and lower vs. college degree and above), injury location (tetraplegia vs. paraplegia), severity (incomplete vs. complete), and time post injury (<1 year vs. >1 year). Items were flagged when the probability associated with the χ^2^ test was <0.01 and the effect size measures (McFadden's pseudo *R*^2^) were greater than 0.02, a small but non-negligible effect. After reviewing item content, we removed items iteratively to retain the best fitting items and allow for the most precise estimation. We repeated the analytic steps until an acceptable solution was attained that confirmed a unidimensional model and no misfitting items or DIF were present.

#### Assessment center programming and short form selection

The IRT parameters for the final set of retained items were then used to program a CAT version of the SCI-QOL Psychological Trauma item bank on the Assessment Center™ platform^48^ (available at www.assessmentcenter.net). Final item parameters were also combined with clinical/expert input to create a static short form (SF) version of the measure, which can be administered by paper and pencil or via Assessment Center.™

## Results

### Patient characteristics

We administered the Psychological Trauma and other SCI-QOL item pools related to emotional health (e.g. Depression, Positive Affect & Well Being, Resilience) to a sample of 716 individuals with SCI. Table 1 provides the sociodemographic and injury-related information.

**Table 1:** Calibration sample – participant characteristics

Variable	*N* = 716 Mean (SD), N (%)
Age (years)	43.0 (15.3)
Age at injury (years)	36.1 (16.8)
Sex	
Male	558 (78%)
Female	158 (22%)
Ethnicity	
Hispanic	81 (11%)
Non-Hispanic	631 (88%)
Not reported/Refused	4 (1%)
Race	
Caucasian	505 (70%)
African-American	125 (17%)
Asian	8 (1%)
American Indian/Alaska Native or Native Hawaiian/Pacific Islander	7 (1%)
More than one race	9 (1%)
Other	49 (7%)
Not provided/Refused	4 (1%)
Time Since Injury	7.1 (10.0)
<1 year post injury	195 (27%)
1–3 years post injury	186 (26%)
>3 years post injury	335 (47%)
Diagnosis	
Paraplegia Complete	182 (25%)
Paraplegia Incomplete	143 (20%)
Tetraplegia Complete	157 (22%)
Tetraplegia Incomplete	230 (32%)
Unknown	4 (0%)
Cause of injury	
Motor vehicle accident	241 (34%)
Fall	164 (23%)
Violence	83 (12%)
Diving	57 (8%)
Other sports	45 (6%)
Motorcycle/ATV/Dirt Bike	29 (4%)
Medical/Surgical accident	27 (4%)
Bicycle accident	9 (1%)
Other	49 (7%)
Not reported	12 (2%)

### Preliminary analysis and item removal

We conducted CFA iterations for the 31-item Psychological Trauma item pool. After each iteration we examined item content, model fit, internal consistency (Cronbach's α), corrected item-total correlations, excessive missing data (missing responses for greater than 5 items), sparse cells (fewer than 5 responses), and violations of monotonicity. After the first iteration we removed 6 items that exhibited local item dependence; some items also demonstrated misfit (significant S-X^2^ statistic), 2 items; low item-total correlation, 4 items; and category inversion, 3 items.

Three of the 25 remaining items demonstrated local dependence or low item-total correlations. DIF analysis on the 22-item set indicated that one item, ‘*I broke into a sweat when I thought about my accident*,’ exhibited DIF for level of education and was removed. Finally, two items has sparse data (*n* < 5) in category 1 (Never) which would have resulted in collapsed categories for those items (i.e. 4 instead of 5) so they were removed from the bank. The following results are based on the final 19-item set.

### Dimensionality

The CFA fit to a unidimensional model was good to excellent (CFI=0.952; RMSEA=0.061). For the majority of items, *R*^2^ values were greater than 0.30, however for 1 item the *R*^2^ values was less than 0.30 (Trauma_21=0.283). While no item pairs met our criteria for LID (residual correlations >|0.20|), two item pairs did have residual correlations >|0.15|. They were the following pairs: *I avoided reminders of how my injury occurred* (Trauma_33) WITH *I was frightened by sudden noises* (Trauma_24) (*r* = –0.157); and *I was nervous when something reminded me of the accident* (Trauma_19) WITH *I felt trapped* (Trauma_13) (*r* = –0.164).

### IRT parameter estimation and model Fit

As seen in Table 2, item slopes ranged from 1.16 to 2.55 and thresholds ranged from –0.78 to 3.44.

**Table 2:** SCI-QOL Psychological Trauma items and item bank parameters

Item ID	Item stem	Item response theory calibration statistics				
		Slope	Threshold 1	Threshold 2	Threshold 3	Threshold 4
Trauma_30	I was afraid in crowds	1.57421	0.95648	1.72136	2.64405	3.35520
Trauma_33	I avoided reminders of how my injury occurred	1.44828	0.09983	0.81558	1.65866	2.24291
**Trauma_19**	**I was nervous when something reminded me of the accident**	**2.19179**	**0.44776**	**1.10091**	**1.90590**	**2.44482**
**Trauma_9**	**I felt emotionally numb**	**2.00586**	**0.25498**	**0.84766**	**1.97258**	**2.79269**
**Trauma_7**	**I had thoughts that were frightening**	**2.25811**	**0.39029**	**1.16919**	**2.22455**	**2.94328**
**Trauma_24**	**I was frightened by sudden noises**	**1.23673**	**0.51372**	**1.45476**	**2.64285**	**3.42974**
**Trauma_4**	**I had upsetting thoughts about the event of my injury**	**2.52436**	**0.14724**	**0.78693**	**1.69816**	**2.37833**
Trauma_11	I avoided sharing my emotions about the accident with other people	1.67124	0.20897	0.76516	1.60297	2.18457
**Trauma_3**	**I saw parts of the accident happen again while I was awake**	**1.43004**	**1.16154**	**1.66489**	**2.68595**	**3.33866**
Trauma_31	I had difficulty concentrating	1.53309	−0.39576	0.45743	2.08463	3.02962
Trauma_32	I was afraid to be left alone	1.64607	0.80443	1.53908	2.56070	3.50183
Trauma_18	I avoided making plans for the future	1.78005	0.35429	0.92148	1.82906	2.39963
Trauma_14	I felt that most people couldn't be trusted	1.19714	0.24197	1.24122	2.40163	3.18524
Trauma_13	I felt trapped	1.91667	0.12041	0.74675	1.53474	2.25364
Trauma_21	I was watchful for anything bad that might happen	1.17283	−0.77668	0.18868	1.30608	2.08346
Trauma_5	I thought about death	1.36813	0.26076	1.10668	2.24075	3.07388
Trauma_8	I avoided things that reminded me of the event that injured me	2.06409	0.67850	1.21361	1.82160	2.23386
**Trauma_22**	**I felt jumpy**	**2.04219**	**0.46488**	**1.25980**	**2.32637**	**3.27630**
**Trauma_25**	**I felt stunned by everything that's happened to me**	**2.40511**	**0.13091**	**0.83137**	**1.70700**	**2.23875**

*Context for all items was: ‘In the past 7 days.’ Response set was: 1 = Never/2 = Rarely/3 = Sometimes/4 = Often/5 = Always.

**Bold font** indicates the items selected for the short form 8a. Items and parameters copyright © 2015 David Tulsky and Kessler Foundation. All Rights Reserved. Scales should be accessed and used through the corresponding author or http://www.assessmentcenter.net. Do not modify items without permission from the copyright holder.

**Figure 1:**
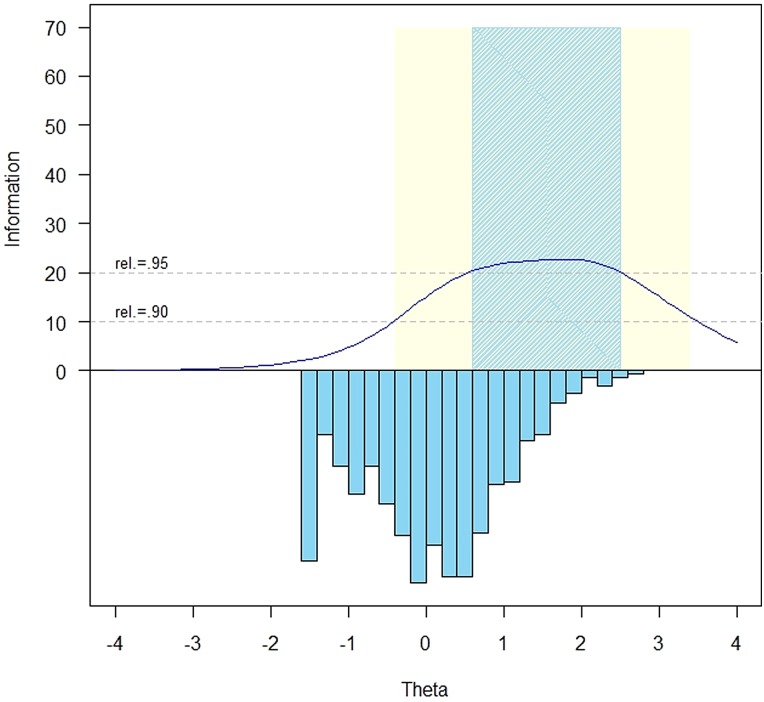
SCI-QOL Psychological Trauma Item Bank Information and Precision.

**Figure 2:**
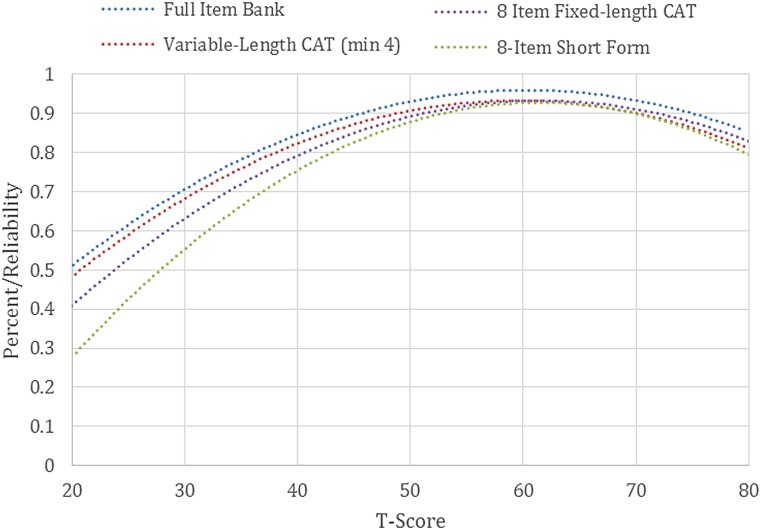
SCI-QOL Psychological Trauma: Measurement reliability by T-score and Assessment Method. Note: CAT=Computer Adaptive Testing. Scores simulated from calibration data. Note: We have not included a curve for the variable-length CAT with a minimum of 8 items as it appears essentially identical to the curve for the 4-item-minimum CAT.

The measurement precision in the theta range between 0.6 and 2.5 is roughly equivalent to a classical reliability of 0.95 or better (Figs. 1 and 2).

The S-X^2^ model fit statistics were examined using the IRTFIT^49^ macro program. All items had adequate or better model fit statistics (P > 0.05) with marginal reliability equal to 0.89.

### Differential item functioning

Nine items were flagged for DIF in at least one category based on the χ^2^ test; however, when the effect size measures were examined, the DIF was negligible in all cases.

### Short form selection and mode of administration

We programmed the 19 items and parameters into the Assessment Center^SM^^48^ platform, which enables the bank to be used as a CAT or downloaded as a static short form. In addition, researchers may also wish to select individual items based on item content and parameters to create a customized short form.

We compared the reliability of the full 19-item bank with the 8-item short form, a variable-length CAT with the Assessment Center default parameters (i.e. minimum # items to administer = 4, maximum number of items to administer = 12, maximum standard error = 0.3), a variable-length CAT with a minimum of 8 items (and the same maximum, 12), and a CAT fixed to 8 items (i.e. length of short form) to examine the level of measurement precision and error across modes of administration. Table 3 presents measures of central tendency and dispersion for the various administration modes.

**Table 3:** Accuracy of psychological trauma variable- and fixed-length CAT and 8-Item short form: correlations with full-bank Score

Mode	*N*	# Items Admin			Max	%Max	%Min	Correlation with full bank
		Mean	SD	Min				(19 Items)
Variable- length CAT (min 4/max 12)	716	10.07	2.4	5	12	54.2	1.1	0.98
Variable-length CAT (min 8)	716	10.44	1.8	8	12	54.2	32.4	0.99
8-Item fixed-length CAT	716	8	0	8	8	n/a	n/a	0.97
8-Item short form	716	8	0	8	8	n/a	n/a	0.95

As expected, the full bank demonstrated the highest level of precision (Fig. 2). Measures from all modes of administration correlate highly with the full bank, with the 8-item minimum variable-length CAT demonstrating the highest level of precision (0.99 correlation with full bank, see Table 3) and the short form the least (0.95). Over 50% of the sample received the maximum number of items (i.e. 12), indicating that a large portion of the sample responded indicating no (or very few) trauma symptoms (Fig. 2). Since these individuals were at the floor of the trait distribution (i.e. where the reliability of trait estimates is lowest), the CAT did not reach a low enough standard error value to discontinue before the maximum number of items. While many of the other SCI-QOL CATs have an average number of items administered of 6–7, the average number of items administered in the Psychological Trauma CAT is just over 10. For this reason, the variable-length CATs perform better than the short form or 8-item fixed length CAT since on average the variable-length CATs are administering 2 additional items. We recommend administering the SCI-QOL Psychological Trauma bank as a CAT with a minimum of 4 items and a maximum of 12 items to maximize measurement precision while limiting response burden.

In some cases, however, it is neither possible (i.e. internet unavailable) or practical (i.e. laptop/tablet computer equipment beyond budget of project) to administer items in this way. As such, and as with all other SCI-QOL item banks, the project investigators utilized psychometric and clinical input to develop a fixed, 8-item ‘short form’ version of the item bank. Short forms may be administered directly within Assessment Center, or may be downloaded from Assessment Center for administration by paper and pencil or an alternate data capture platform or system.

### Scoring

SCI-QOL Psychological Trauma scores are standardized on a T metric, with a mean of 50 and a standard deviation of 10, based on the SCI-QOL calibration data (i.e. a mean of 50 reflects the mean of an SCI population rather than the general population). All CAT administrations of the SCI-QOL Psychological Trauma item bank are automatically scored by Assessment Center. When administering the short form, whether via Assessment Center, paper and pencil, or another data capture platform, an individual must complete all 8 component items in order to receive a valid scaled score. The raw score for the short form is computed by simply summing the response scores for the individual component items. The T-score and associated standard error for each raw score value are located in Table 5.[Table JSCM-D-15-00010TB4]

**Table 4 JSCM-D-15-00010TB4:** Breadth of coverage for psychological trauma variable-length CAT, fixed-length CAT, 8-item short form, and full item bank

Mode	*N*	T-Score				Standard error	
		Mean±SD	Range	% Ceiling	% Floor	Mean±SD	Range
Variable-length CAT (min 4)	716	50.03 ± 9.22	33.85–76.70	0.14	8.94	0.359 ± 0.099	0.280–0.597
Variable-length CAT (min 8)	716	50.02 ± 9.22	33.85–76.70	0.14	8.94	0.354 ± 0.103	0.246–0.597
8-Item fixed-length CAT	716	50.12 ± 9.01	35.39–79.08	0.14	11.73	0.382 ± 0.114	0.246–0.616
8-Item short form	716	50.13 ± 8.75	38.40–78.00	0.14	21.09	0.412 ± 0.124	0.260–0.620
Full bank	716	49.98 ± 9.43	33.10–76.70	0.14	8.66	0.312 ± 0.113	0.190–0.580

**Table 5:** T-score lookup table for SCI-QOL psychological trauma 8-Item Short Form (SF8a)

Raw score	Scaled score	Standard error
8	38.4	6.2
9	43.9	4.8
10	46.1	4.6
11	48.5	4.2
12	50.2	4.1
13	51.9	3.7
14	53.3	3.6
15	54.6	3.4
16	55.9	3.4
17	57	3.3
18	58.2	3.3
19	59.2	3.3
20	60.3	3.3
21	61.4	3.3
22	62.4	3.3
23	63.4	3.3
24	64.4	3.3
25	65.5	3.3
26	66.5	3.3
27	67.5	3.3
28	68.6	3.3
29	69.6	3.3
30	70.7	3.3
31	71.8	3.3
32	72.9	3.4
33	74.1	3.4
34	75.3	3.5
35	76.5	3.5
36	77.9	3.6
37	79.3	3.7
38	80.9	3.9
39	82.7	3.9
40	85.2	4.1

### Reliability

As a part of the reliability study described in the Tulsky *et al*.^42^ methods paper in this issue, we compared Psychological Trauma scores at Baseline with those from the 1–2 week retest assessment. In a sample of 245 individuals with SCI, Pearson's *r* = 0.84 and ICC (2,1) = 0.84 (95% CI = 0.80 to 0.88).

## Discussion

SCI presents considerable challenges to physical, psychological and social well-being. Longitudinal studies have reported that overall rates of injury-related distress among individuals with SCI changes little over time, and that individuals displaying clinically significant symptoms of emotional distress in the weeks immediately following injury are significantly more likely to do so in the future, even as far out as 10 years, highlighting the need for early interventions.^50,51^

The measurement of psychosocial issues following traumatic injury has improved dramatically over the past 10 years. Measures in areas such as depression, alcohol use/abuse, and social participation have all become central variables in clinical research. Unfortunately, though, measurement of anxiety in general and especially in the more extreme form as PTSD, has received less attention by the medical community.

Traumatic SCI is, by definition, the type of trauma that could cause post-traumatic stress. There seems to be a disconnect between the high rates of PSTD (i.e. between 10–40%)^52^ and the fact that it has not been studied more in this population, which leads us to conclude that this is a very understudied area. More research is necessary to determine how to prevent and treat PTSD and also to determine whether this is in response to the event of prior trauma, to the event of SCI, or to the physical disabilities caused by SCI. More research is needed to determine whether these psychological reactions to trauma are universal in persons with SCI. Also, additional research can determine whether the SCI-QOL Psychological Trauma item bank is sensitive to identify individuals who are at risk for PTSD.

It is for all of these reasons that the SCI-QOL Psychological Trauma item bank was developed to measure the constellation of psychological symptoms and issues related to the traumatic and stressful event of an SCI. We removed misfitting items systematically based on psychometric and clinical criteria and the final SCI-QOL Psychological Trauma item bank contains 19 items; users have the option of using an 8-item short form or CAT. This measure has been developed to help fill the research gap related to SCI and PTSD and the item bank is intended to serve as an SCI-specific indicator of clinical or subclinical levels of psychological consequences of trauma/post-traumatic stress for SCI research. In the future, it would be helpful to evaluate the clinical potential of this scale in assisting with the formal diagnosis of PTSD and to move the SCI-QOL Psychological Trauma scale from a research tool to a clinical measurement instrument. Several new areas of research are recommended. These would include establishing minimally clinically important differences^53^ and developing clinically meaningful standards or scoring cut-points.^54^ Additionally, we should view reactions to trauma and PTSD as changing states. It will be necessary to examine clinical trajectories and patterns of change over time^55,56^ and develop clinically meaningful standards of these change scores. We should be able to detect when the change represents a clinically meaningful state that health care providers should attend to and implement treatment procedures. An additional area that is currently understudied is the impact of concomitant TBI with SCI, especially given the potential for the confounding of TBI sequelae with PTSD symptomatology. We recommend further research in individuals with concomitant SCI and TBI, perhaps including development of separate clinical guidelines for use in this population. As described in the Introduction, the impact of the psychological consequences of the SCI and its related traumatic event is an understudied area. The item bank described in this manuscript represents a way to diagnose psychological trauma and has the potential to greatly facilitate the future study of trauma/PTSD in individuals with SCI.

## Conclusion

With this paper, we offer the SCI research community a new tool to identify individuals’ functioning related to the psychological consequences of trauma. We developed this scale using qualitative feedback from individuals with SCI to ensure a patient-centered approach to assessment and then we tested the items in a large sample of individuals with SCI and state-of-the-art quantitative measurement techniques. We have developed a 19-item bank that may be administered using a subset of these items as either a CAT or fixed-length short form. This new tool should help fill the gap in measurement of this important psychosocial outcome in SCI research.

## Disclaimer statements

**Contributors** All authors have contributed significantly to the design, analysis and writing of this manuscript. The contents represent original work and have not been published elsewhere. No commercial party having a direct financial interest in the results of the research supporting this article has or will confer a benefit upon the authors or upon any organization with which the authors are associated.

**Funding** This study was supported by National Institutes of Health grant number 5R01HD054659 (Eunice Kennedy Shriver National Institute of Child Health and Human Development/National Center on Medical Rehabilitation Research and the National Institute on Neurological Disorders and Stroke).

**Conflicts of interest** All SCI-QOL items and parameters are © 2015 David Tulsky and Kessler Foundation. All rights reserved. All items are freely available to the public via the Assessment Center platform (www.assessmentcenter.net). There are currently no plans for Dr. Tulsky or Kessler Foundation to profit from the use of the SCI-QOL instrument.

**Ethics approval** The Institutional Review Board at each site reviewed and approved this project.
